# A Low-Frequency MEMS Piezoelectric Energy Harvesting System Based on Frequency Up-Conversion Mechanism

**DOI:** 10.3390/mi10100639

**Published:** 2019-09-24

**Authors:** Manjuan Huang, Cheng Hou, Yunfei Li, Huicong Liu, Fengxia Wang, Tao Chen, Zhan Yang, Gang Tang, Lining Sun

**Affiliations:** 1 School of Mechanical and Electric Engineering, Jiangsu Provincial Key Laboratory of Advanced Robotics, Soochow University, Suzhou 215123, China; szdxhmj@126.com (M.H.); livehc@126.com (C.H.); liyunfei3321@foxmail.com (Y.L.); chent@suda.edu.cn (T.C.); yangzhan@suda.edu.cn (Z.Y.); lnsun@hit.edu.cn (L.S.); 2 Jiangxi Province Key Laboratory of Precision Drive & Control, Department of Mechanical and Electrical Engineering, Nanchang Institute of Technology, Nanchang 330099, China; tanggang@nit.edu.cn

**Keywords:** MEMS, piezoelectric vibration energy harvester, frequency up-conversion mechanism, impact, PZT thick film

## Abstract

This paper proposes an impact-based micro piezoelectric energy harvesting system (PEHS) working with the frequency up-conversion mechanism. The PEHS consists of a high-frequency straight piezoelectric cantilever (SPC), a low-frequency S-shaped stainless-steel cantilever (SSC), and supporting frames. During the vibration, the frequency up-conversion behavior is realized through the impact between the bottom low-frequency cantilever and the top high-frequency cantilever. The SPC used in the system is fabricated using a new micro electromechanical system (MEMS) fabrication process for a piezoelectric thick film on silicon substrate. The output performances of the single SPC and the PEHS under different excitation accelerations are tested. In the experiment, the normalized power density of the PEHS is 0.216 μW·g^−1^·Hz^−1^·cm^−3^ at 0.3 g acceleration, which is 34 times higher than that of the SPC at the same acceleration level of 0.3 g. The PEHS can improve the output power under the low frequency and low acceleration scenario.

## 1. Introduction

In recent years, with the rapid development of micro electromechanical systems (MEMSs) and the Internet of things (IoT), various micro wireless sensor nodes (WSNs) have been developed. These nodes are widely used in military surveillance, structural health monitoring, road traffic monitoring, and so on [1,2,3,4]. However, the limited lifetime of traditional batteries restricts the application of WSNs in complex environments and increases the working load of changing the batteries periodically. To overcome this restriction, some environmental energy harvesters dedicated to collect solar, thermal, wind, ocean wave, and vibration energies have been developed [5]. Among these, vibration energy is ubiquitous, such as structural vibrations, human activities, and fluid flows. The mechanical vibration energy can be converted into electrical energy through four transduction mechanisms, which are electromagnetic [6,7], piezoelectric [8,9,10], triboelectric [11,12], and electrostatic [13,14]. Piezoelectric vibration energy harvesters (PVEHs) have received significant attention due to their simple configuration, high energy conversion efficiency, and precision controllability of the mechanical response [15,16,17].

Some piezoelectric materials are widely used in MEMS energy harvesters, which are aluminum nitride (AlN) [18,19], zinc oxide (ZnO) [20,21], and Pb(Zr_x_Ti_1-x_)O_3_ (PZT) [22,23,24,25,26,27]. Among these, PZT has a higher electromechanical coupling coefficient compared with AlN and ZnO. Cui et al. [26] developed a multi-beam energy harvester with a PZT thin-film layer using a sol-gel deposition method. The maximum output power of 16.74 nW was obtained under an acceleration of 1 g and resonant frequency of 1400 Hz. Generally, PZT thin-film deposition requires a specific and complicated fabrication recipe, and the output performance of the PZT thin-film is limited. Therefore, PZT thick-film-based energy harvesters were developed. Xu et al. [28] proposed a screen-printed PZT/PZT thick-film bimorph cantilever for energy harvesting. However, the screen-printed PZT thick films are not dense enough, which means their piezoelectricity is low compared with that of bulk PZT. Thus, preparing a high-quality PZT thick film on silicon (Si) substrate through wafer bonding of bulk PZT has been proposed [29,30,31]. Janphuang et al. [30] demonstrated a wafer-level fabrication process of piezoelectric energy harvester using a spin-on polymetric adhesive WaferBOND as a bonding layer between bulk PZT and Si. The harvester exhibited an average power of 82.4 µW under an excitation of 1 g at 96 Hz. The above studies indicate that the MEMS PVEHs with thinned bulk PZT thick films have the potential for high output performance.

Another challenge for MEMS PVEHs is that the resonant frequencies of piezoelectric cantilevers are higher than most ambient vibration sources. Most of the natural vibration sources are random and at a low-frequency, typically ranging from 30 to 200 Hz [32,33,34]. In order to effectively utilize the low-frequency environmental vibrations, lowering the resonant frequency and widening the operating bandwidth have been the major target for the small-scale PVEHs. The frequency up-conversion mechanisms provided a good solution to address these issues and have aroused great research interest [35,36,37,38]. In general, the frequency up-conversion technologies can be divided into non-impact and impact types. Galchev et al. [36] demonstrated a non-impact piezoelectric generator that utilized a magnetic latching mechanism to convert the ambient low frequency to a higher internal operation frequency. However, the average power of the device was 3.25 µW at 1 g. Improvement of the output power needs to be considered. Jung et al. [39] introduced an energy harvester that uses the snap-through buckling action of a pre-buckled beam for frequency-up conversion instead of magnetic coupling. A maximum output power of 131 µW was generated using a 3 g acceleration. Andò et al. [40] proposed a snap-through buckling based vibrational energy harvester by adopting a flexible buckled beam, which was able to generate power in the excess of 400 µW under an acceleration of 13.35 m/s^2^. However, large accelerations are generally required to drive the beam to induce snap-through buckling, and it is difficult to fabricate the buckled beam with standard technologies. In addition to these non-impact frequency up-conversion approaches, Umeda et al. [37] first demonstrated the impact-based frequency up-conversion approach for energy harvesting by investigating the power transformation of a steel ball impacting on a piezoelectric membrane. Halim et al. [38] proposed a mechanical impact-driven PVEH consisting of two series-connected PZT cantilevers and a flexible driving cantilever. A peak power of 734 µW from two series connecting PZT beams was achieved at the resonant frequency of 14.5 Hz. The impact-driven frequency up-conversion technology effectively increases the output power of the energy harvester at low frequency. Liu et al. [8] developed a PZT thin-film MEMS-based frequency up-converted PVEH system by utilizing the periodic impact between an S-shaped, low-frequency driving cantilever and a straight, high-frequency PZT generating cantilever. The PVEH system realized a low operating frequency under 37 Hz and the volume was very small. However, the maximum output power was only 0.12 µW with a 0.8 g acceleration. So far, there have been few studies on silicon-based PVEH fabricated at the scale of MEMS for harvesting energy from low-frequency vibration through an impact-based frequency up-conversion mechanism.

Therefore, this study has carried out research and discussion targeting a low-frequency MEMS PVEH by using a frequency up-conversion mechanism. First, a new wafer-level micromachining process for fabricating the PZT thick-film cantilever energy harvester was put forward. Then, the piezoelectric energy harvesting system (PEHS) with a low-frequency S-shaped stainless-steel cantilever (SSC) and a high-frequency straight piezoelectric cantilever (SPC) was incorporated. The output performances of the system and the single SPC were investigated and compared by using a vibration control and testing system. The experimental results indicated that the impact-based frequency up-conversion mechanism was able to improve the output performance of the harvester under a low-frequency and low-acceleration vibration environment.

## 2. Design and Simulation

### 2.1. Device Configuration

**Figure 1:**
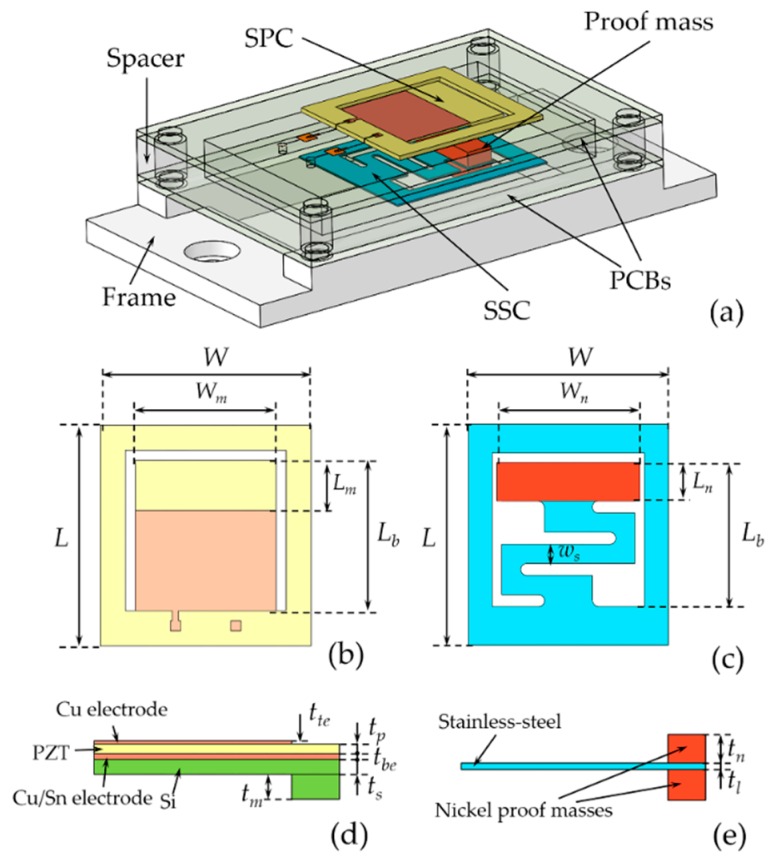
(**a**) 3D schematic of the piezoelectric energy harvesting system. (**b**) Schematic diagrams of the straight piezoelectric cantilever and (**c**) the S-shaped stainless-steel cantilever. (**d**) Sectional views of the piezoelectric cantilever and (**e**) the stainless-steel cantilever.

In order to harvest the low frequency vibration, a PEHS working with a frequency up-conversion mechanism was designed and is shown in Figure 1a. As can be seen, the PEHS was designed as a parallel-cantilever structure, which consisted of a top high-frequency SPC and a bottom low-frequency S-shaped SSC assembled within a predefined space. Figure 1b,c shows the schematic diagrams of the SPC and the SSC. The surface area of the SPC was 15 × 14 mm^2^ and the dimensions of the whole chip was 22 mm × 21 mm × 0.6 mm. The S-shaped structure of the SSC was used to achieve a low-stiffness beam within a small space. At the free end of the SSC, two nickel proof masses were assembled to further reduce the resonant frequency of the cantilever. The SSC was mounted on a piece of printed circuit board (PCB). The SPC was mounted on another piece of PCB, assembled on the top of the SSC. The top and bottom electrode pads of the SPC were connected to the lead interfaces of the PCB using gold thread. A rectangular hollow spacer was fixed between the two PCBs, and the initial gap distance between the SPC and SSC could be adjusted by changing the thickness of the spacer. During the vibration of the PEHS, the frequency up-conversion was realized through the periodic collision between the SSC and SPC. Figure 1d shows the sectional view of the multi-layer SPC. The cantilever consisted of a top Cu electrode layer, a PZT thick film layer, a bottom Cn/Sn electrode layer, a Si supporting layer, and a Si proof mass at the cantilever tip. The thickness of the PZT layer and Si layer were *t_p_* and *t_s_*, respectively. The free end of the cantilever was fixed with a Si mass of thickness *t_m_* and length *L_m_* to reduce the resonance frequency. Figure 1e shows the sectional view of the SSC, where the thickness of the stainless-steel was 100 μm. Table 1 lists the detailed geometric parameters of the SPC and the SSC.

**Figure 2:**
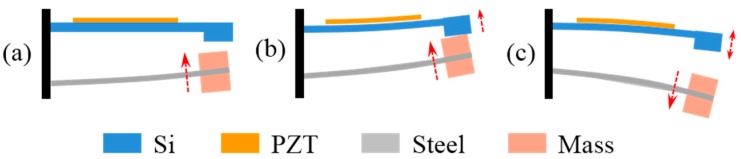
Schematic diagram of the collision process in one cycle: (**a**) approaching, (**b**) impacting, and (**c**) separating states.

Figure 2 shows the collision process in one cycle, which can be divided into three states: approaching, impacting, and separating. Assume that the PEHS is excited by a sinusoidal external vibration, and the frequency of the vibration is close to the resonant frequency of the SSC. In the approaching state, the SSC bends and moves upward to the SPC due to the external force. Since the deformation of the SPC is much smaller than that of the SSC, the SPC would hinder the displacement of the SSC. In the impacting state, the SSC impacts on the SPC and then moves upward together with the SPC. After that, the two cantilevers move downward and separate, then vibrate independently at their own resonant frequency until the next collision. As a result, the low-frequency vibration of the SSC is transformed into the high-frequency resonation of the SPC.

### 2.2. Modal Analysis Using COMSOL

**Figure 3:**
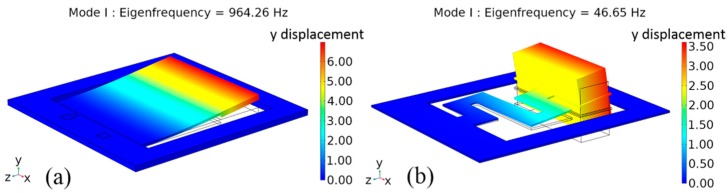
Modal analysis of (**a**) the straight piezoelectric cantilever, and (**b**) the S-shaped stainless-steel cantilever.

The resonant modes of the SPC and SSC were simulated using the finite element analysis software COMSOL 5.4a (Stockholm, Sweden), as shown in Figure 3. In the simulation, since the top and bottom electrode layers in SPC were too thin, which would lead to an increase in the calculation amount of the mesh division, the simulation model was simplified. The materials of the SPC in the model were defined as Si and PZT-5H. Meanwhile, the materials of the SSC were defined as stainless-steel and nickel. Figure 3a,b show the first-order vibration mode shapes (mode I) and eigenfrequencies of the SPC and SSC, respectively. The simulated resonant frequencies of the high-frequency SPC and the low-frequency SSC were 964.26 and 46.65 Hz, respectively. As can be seen, the maximum y displacements of the SPC and SSC both occurred at the end of the cantilever beam.

## 3. Micro Fabrication Process

**Figure 4:**
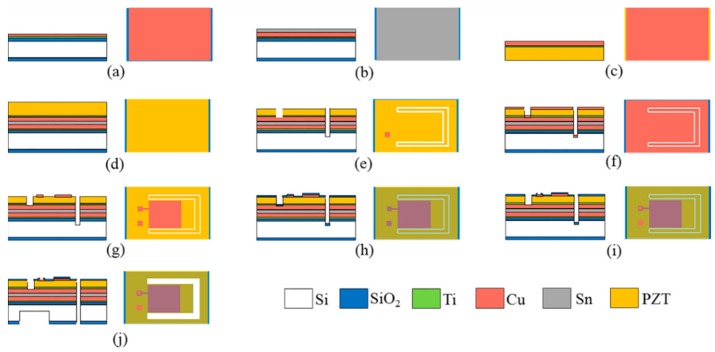
Schematic illustration of the fabrication process of the straight piezoelectric cantilever. (**a**) Sputtering Ti/Cu seed layer and (**b**) electroplating Cu/Sn layer on Si substrate. (**c**) Sputtering Ti/Cu seed layer and electroplating Cu layer on PZT wafer. (**d**) Bonding PZT wafer and Si substrate together. (**e**) Laser cutting the electrode pads and thinning the bulk PZT by mechanical lapping. (**f**) Sputtering Cu top electrode layer. (**g**) Top electrode patterning through lithography and IBE process. (**h**) Depositing SiO_2_ by PECVD. (**i**) Welding spots patterning through lithography and RIE process. (**j**) Si proof mass patterning and backside DRIE to release cantilever.

As mentioned above, the PZT thick-film SPC was a PZT/Si composite structure with a Si proof mass on the free end. A schematic illustration of the wafer-level micro fabrication process of the SPC is shown in Figure 4, which mainly included the bonding of bulk PZT and Si wafer, thinning of PZT thick film, electrodes preparation, proof mass etching, and cantilever releasing.

The fabrication process began with a 4-inch double-side-polished bare Si wafer with a thickness of 500 μm. Then, a 500 nm thick silicon dioxide (SiO_2_) layer was grown on both sides of the Si wafer using plasma enhanced chemical vapor deposition (PECVD), which served as a mask layer during the etching process. A Ti (20 nm)/Cu (50 nm) seed layer was sputtered on the cleaned Si substrate through magnetron sputtering, as shown in Figure 4a. The seed layer helped to enhance the adhesion of the metal to the Si wafer during the next electroplating. A 5-μm thick Cu layer and a 4.5-μm thick Sn layer were electroplated onto the Si wafer (Figure 4b). Another Ti (20 nm)/Cu (50 nm) seed layer was sputtered on the cleaned 4-inch bulk PZT wafer with a thickness of 400 μm, followed by a 5-μm thick Cu layer electroplated onto the PZT wafer (Figure 4c). Then, the PZT wafer and Si substrate were bonded together by means of Cu-Sn-Cu eutectic bonding (Figure 4d). The metal bonding layer also functioned as the bottom electrode layer. After bonding, the shape of the cantilever and bottom electrode pads were cut on the surface of the PZT wafer using laser cutting. The cutting depth of the bottom electrode pads should be exactly stopped at the metal bonding layer, and the cutting depth of the cantilever boundary should be deeper than the binding layer. Then, the bulk PZT was thinned from 400 μm to 65 μm using mechanical lapping (Figure 4e). Subsequently, a 1-μm thick Cu layer as the top electrode was sputtered onto the polished surface of the PZT using magnetron sputtering (Figure 4f). The top Cu electrode was patterned using ultraviolet (UV) lithography and etched using ion beam etching (IBE) (Figure 4g). A 500-nm thick SiO_2_ layer was deposited on the PZT surface using PECVD. This layer was used to prevent the top electrode Cu from being oxidized in the air (Figure 4h). Next, the welding spots of the top and bottom electrodes were patterned using UV lithography and the SiO_2_ layer was etched using reactive ton etching (RIE) (Figure 4i). Finally, the structure of the Si proof mass on the backside of the Si wafer were patterned using UV lithography, and then etched through the 500-nm thick SiO_2_ using RIE. After the oxide layer was etched, a deep reactive ion etching (DRIE) dry etching process was utilized to ultimately release the cantilever (Figure 4j).

**Figure 5:**
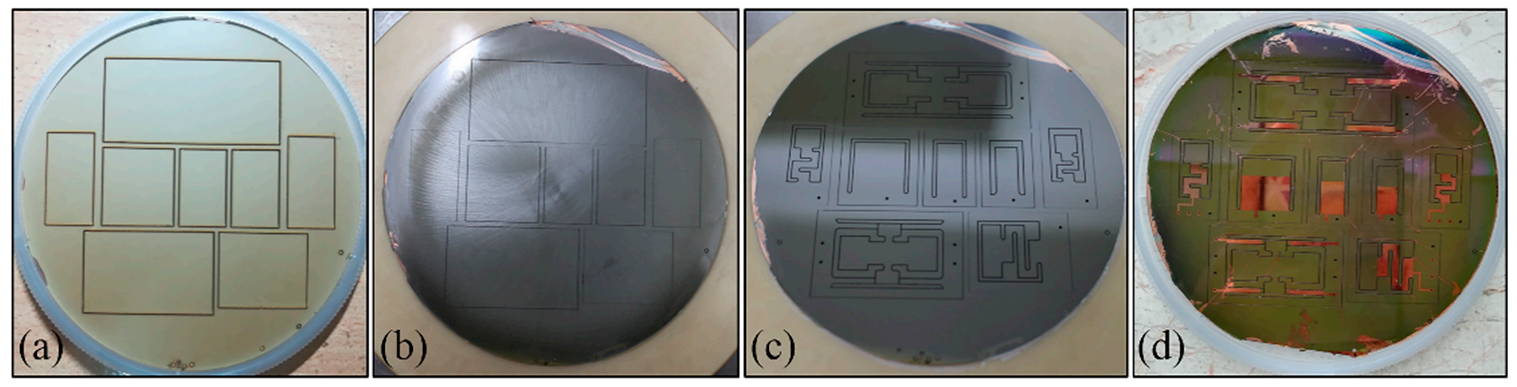
Photographs of the wafer after being (**a**) bonded, (**b**) thinned, (**c**) polished, and (**d**) etched.

In the above MEMS process, a new bonding method for the bulk PZT and Si wafer was proposed, and the metal bonding layer was employed as the bottom electrode as well, which reduced the step of fabricating the bottom electrode and simplified the process. Preparation of the PZT thick film on the Si substrate was the key technique. It mainly consisted of two steps, which were bonding the bulk PZT wafer with the Si wafer and thinning the bulk PZT to the desired thickness, as shown in Figure 4d,e. Figure 5a shows the photograph of the wafer after being bonded. The Cu-Sn-Cu eutectic bonding method was developed to bond the bulk PZT and Si wafer at 270 °C for 30 min. Since the melting point of Sn is 231.9 °C, in order to ensure that Cu and Sn were sufficiently mutually fused, the bonding temperature should be higher than the melting point of Sn, which produces a high bonding strength. However, the Curie temperature of the PZT material is 295 °C [32]. A high bonding temperature may result in a reduction of the voltage output performance of the PZT thick film, in addition to forming large thermal stresses in the PZT layer. To prevent the PZT layer from cracking due to excessive thermal stress during the thinning process, some grooves were laser-cut on the surface of the PZT layer before the thinning to release the thermal stress in the wafer. In order to further solve these problems, subsequent research should be focused on the development of low-temperature, high-strength bonding methods. The photograph of the wafer after being thinned is shown in Figure 5b. It can be seen that due to the uneven thickness of the bonding layer, the PZT layer on one side of the wafer was completely worn through during the mechanical lapping. Therefore, when placing the device structure on the wafer, it should be placed in the middle as much as possible. Subsequently, in order to facilitate the patterning of the top Cu electrode, the shape of the device was cut out using laser cutting on the surface of the PZT layer. The PZT layer was then polished, and the polished wafer is shown in Figure 5c. Figure 5d shows the photograph of the wafer after being etched, where the shape of the top electrode was patterned using lithography and IBE. The advantage of this new wafer level MEMS process is the ability to simultaneously fabricate PZT thick film energy harvesters of different structures, reducing manufacturing costs and enabling mass production.

**Figure 6:**
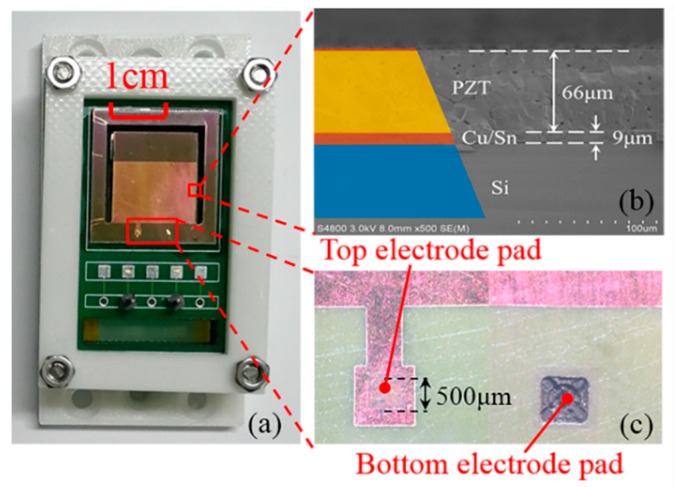
(**a**) Photograph of the PEHS. (**b**) Cross-section SEM image of the piezoelectric cantilever. (**c**) Photomicrograph of top and bottom electrode pads.

The photograph of the PEHS prototype is shown in Figure 6a. Figure 6b shows the cross-section scanning electron microscope (SEM) image of the SPC. The multilayer piezoelectric cantilever consists of a 65-μm PZT layer with a 1-μm Cu electrode layer coated on it, a 9-μm intermediate Cu-Sn-Cu bonding layer, and a 200-μm Si substrate. The thickness of the cantilever was controlled using a DRIE process to about 275 μm. Figure 6c shows the photomicrograph of the top and bottom electrode pads. The bottom electrode pad was obtained using laser cutting. The area of the electrode pad was 0.5 mm × 0.5 mm.

## 4. Experimental Results and Discussion

**Figure 7:**
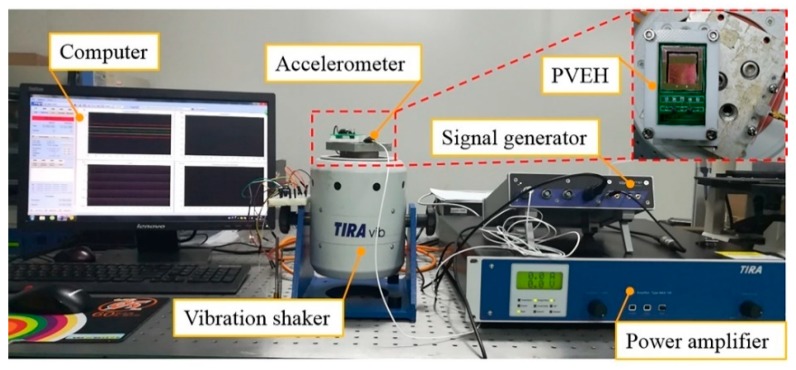
Experimental setup.

Figure 7 shows the experimental setup for the dynamic characterization of the fabricated device. The PVEH prototype (see Figure 6a) was mounted onto a TIRA vibration exciter (TIRA GmbH, Thuringia, Germany) which can generate different external sinusoidal excitations. The sinusoidal excitation signal of the shaker was created using the signal generator and adjusted using the power amplifier. An accelerometer (model 3035BG, DYTRAN, Los Angeles, CA, USA) was fixed on the vibration shaker to monitor the excitation acceleration. The electrical output of the device was recorded via dynamic signal analyzer software on the computer. In this study, in order to verify the effectiveness of the frequency up-conversion mechanism, the output performance of the SPC and the assembled PEHS were tested using a frequency up-sweep method and compared.

### 4.1. The Output Performance of the SPC

**Figure 8:**
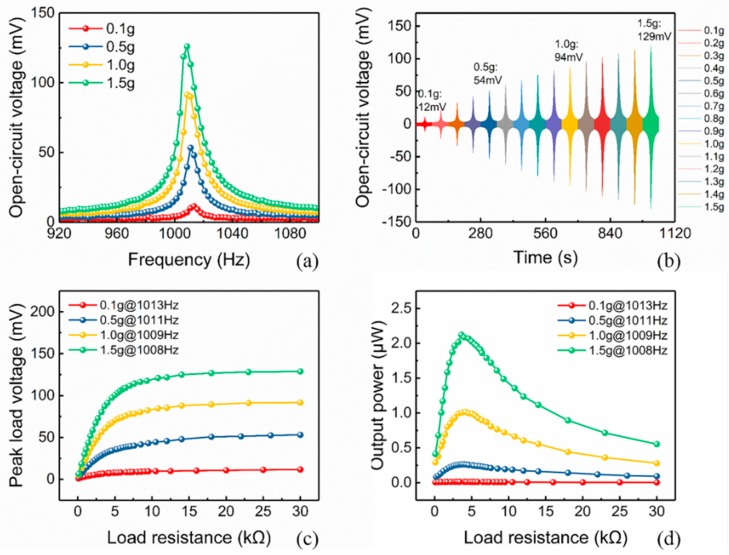
Output performance of the single piezoelectric cantilever. (**a**) The open-circuit voltage versus frequency at different acceleration levels. (**b**) The open-circuit voltage output versus time at various accelerations from 0.1 g to 1.5 g. (**c**) The peak load voltage, and (**d**) the maximum instantaneous output power versus load resistance at accelerations of 0.1 g, 0.5 g, 1.0 g, and 1.5 g.

First, Figure 8a shows the open-circuit voltage at various frequencies from 920 Hz to 1100 Hz under different acceleration levels. It can be seen that the resonant frequencies of the SPC gradually decreased as the acceleration increased; when the applied accelerations were 0.1 g, 0.5 g, 1.0 g, and 1.5 g, the resonant frequencies were 1013 Hz, 1011 Hz, 1009 Hz, and 1008 Hz, respectively. This was because of the nonlinear change in the Young’s modulus of PZT under a large stress [24,41]. According to the previous modal analysis using COMSOL, the first order resonant frequency of SPC was expected to be 964.26 Hz, which was close to the experimental results. The discrepancy between the simulation and the experimental results may be due to the simplification of the simulation model.

Figure 8b shows the open-circuit voltage output versus time at accelerations from 0.1 g to 1.5 g. It is clear that the peak open-circuit voltage increased with the increase of the acceleration, which were 12 mV, 54 mV, 94 mV, and 129 mV at the accelerations of 0.1 g, 0.5 g, 1.0 g, and 1.5 g, respectively. To determine the maximum output power of the SPC with the optimal resistance, the voltage output signal was connected to a varying resistor to obtain the relationship between load resistance and output voltage under different vibration conditions. The instantaneous power delivered by the energy harvester can be expressed as:

(1) $$P=V_{p}^{2}/R$$

where $V_{p}$ is the voltage across the load, and R is the value of the external load resistance.

Figure 8c shows the peak load voltage ($V_{p}$) of the SPC versus the load resistance at different applied acceleration amplitudes of 0.1 g, 0.5 g, 1.0 g, and 1.5 g. Comparing the load voltages under different accelerations, it can be seen that the load voltage increased as the acceleration increased. Furthermore, under a constant acceleration condition, the load voltage clearly increased with the increasing of the load resistance. Based on Equation (1), the maximum output power for different load resistances was calculated and depicted in Figure 8d. A maximum output average power appeared at the optimal matched load resistance, which should be the same as the internal resistance of the device. The value of the optimal matched resistance was related to the acceleration amplitude. For instance, the optimal load resistance under 0.5 g, 1.0 g, and 1.5 g acceleration conditions were 4.2 kΩ, 4.0 kΩ, and 3.6 kΩ, respectively. A conclusion can be drawn that within a certain range of acceleration, the optimal load resistance decreased gradually with the increasing acceleration. As shown in Figure 8d, the maximum output power was 2.12 μW and occurred at the quite high resonance of 1008 Hz and acceleration of 1.5 g.

### 4.2. The Output Performance of the PEHS

**Figure 9:**
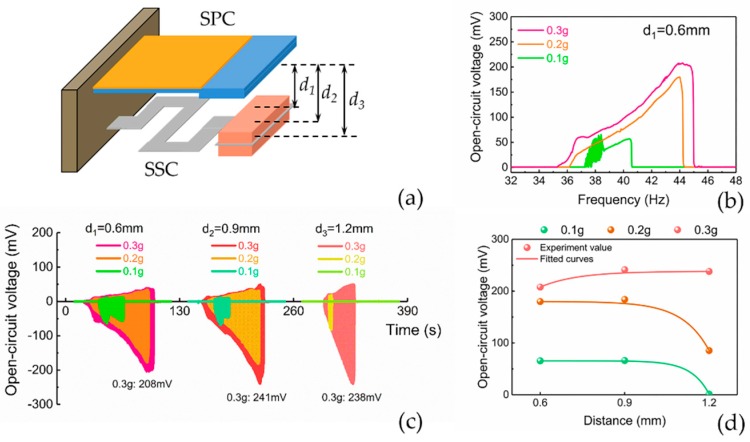
(**a**) The simplified models of the piezoelectric energy harvesting system. (**b**) The open-circuit voltage in the frequency domain at various acceleration levels under the gap of 0.6 mm. (**c**) The open-circuit voltage output in the time domain at various accelerations under different gaps of 0.6 mm, 0.9 mm, and 1.2 mm. (**d**) Relationship between the maximum open-circuit voltage and the initial gap distance.

Figure 9a shows the simplified 3D models of the PEHS. The resonant frequency of the SSC was about 40 Hz, obtained using frequency sweep test, which was close to the simulated resonant frequency of 46.65 Hz in COMSOL. Some factors, such as the gap distance between the SPC and the SSC, as well as the vibration acceleration amplitudes, have an influence on the output performance of the PEHS. In order to investigate the effects of the gap distance on the output performance of the PEHS, the output voltages under three gap distance values *d*_1_, *d*_2_, and *d*_3_ were tested using up-sweep. The gap distance should be limited such that the SSC can impact the SPC during low-frequency vibration. However, the gap cannot be equal to zero, because the high-frequency SPC would limit the ability of the low-frequency driving beam SSC to respond to an external low acceleration and low frequency excitation [42]. Here the values of *d*_1_, *d*_2_, and *d*_3_ were set as 0.6 mm, 0.9 mm, and 1.2 mm, respectively.

The measured open-circuit voltage of the PEHS against operating frequencies at various acceleration levels under the gap distance of 0.6 mm is shown in Figure 9b. The maximum open-circuit voltages at the acceleration of 0.1 g, 0.2 g, and 0.3 g were 64 mV, 180 mV, and 208 mV, respectively. It was observed that under a certain gap and a certain acceleration condition, the open-circuit voltage output increased steadily as the operating frequency increased and then fell abruptly. Figure 9b shows that at an acceleration of 0.3 g, the half-bandwidth of the PEHS at a gain of 0.5 was approximately 5 Hz (from 40 Hz to 45 Hz). The reason for the wide operating bandwidth was that the SSC impacted with the SPC, resulting in a hindrance of the motion of the SSC. The frequency response of the SSC deviated from its normal linear behavior and exhibited nonlinearity in the overall stiffness of the SSC [43].

Figure 9c shows the time domain open-circuit voltage output waveforms of the PEHS under three different gaps at accelerations of 0.1 g, 0.2 g, and 0.3 g. The maximum peak voltages were approximately 208 mV, 241 mV, and 238 mV for the distances of 0.6 mm, 0.9 mm, and 1.2 mm, respectively, at an acceleration of 0.3 g. The maximum voltage of the waveforms for a 0.9 mm gap was higher than those under the other gap conditions. However, considering the bandwidth of the voltage waveforms under the three gap conditions, the maximum bandwidth was achieved under a small gap distance of 0.6 mm. It was observed that under the large gap of 1.2 mm, the SSC could not hit the SPC at all at the low vibration acceleration of 0.1 g. Figure 9d shows the fitted curves of the maximum output voltages under three different gap distances at a certain acceleration amplitude, which indicates the relationship between the output performance of the PEHS and the gap distance. Under the condition that the base acceleration was 0.3 g, the voltage continued to increase slightly as the distance increased from 0.6 mm to 1.2 mm. However, the tendency of the voltage curve at 0.1 g and 0.2 g was to increase slightly over a certain distance range and then decrease. The maximum voltage of 238 mV appeared at 0.3 g for a distance of 0.9 mm. It can be inferred that at a certain vibration acceleration, there may exist an optimal distance under which the maximum output voltage can be obtained.

**Figure 10:**
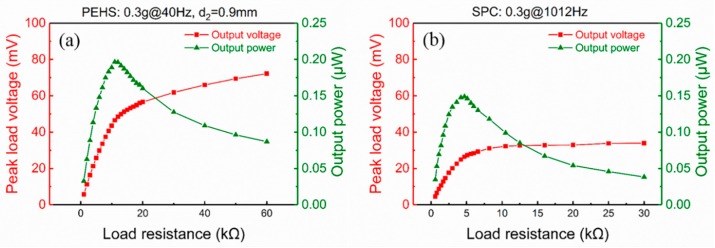
The output load voltage and power versus load resistance of (**a**) the piezoelectric energy harvesting system at 40 Hz, and (**b**) the single straight piezoelectric cantilever at 1012 Hz with a 0.3 g acceleration.

Figure 10a shows the peak load voltage and the maximum output power of the PEHS versus load resistance at a 0.3 g acceleration under the resonant frequency of 40 Hz. The gap distance was 0.9 mm. With the increasing of the load resistance, the load voltage clearly increased, while the corresponding power increased to a maximum value and then decreased. The maximum value of the output power was 0.2 μW at the optimal load resistance of 11 kΩ. The power density of the PEHS normalized by the input acceleration and frequency was 0.216 μW·g^−1^·Hz^−1^·cm^−3^. To verify the effectiveness of the PEHS, the load voltage output of the SPC was also measured at the same acceleration level of 0.3 g under its resonant frequency of 1012 Hz. The peak load voltage and the calculated output power of the SPC for different load resistances is shown in Figure 10b. The tendencies of the voltage and power curves of the SPC were the same as that of the PEHS. However, both the maximum load voltage and the maximum power of the SPC were smaller than those of the PEHS. The maximum output power of the SPC at 0.3 g was 0.15 μW, and the corresponding optimal load resistance was 4.7 kΩ. The normalized power density of the SPC was 0.006 μW·g^−1^·Hz^−1^·cm^−3^ at a vibration acceleration of 0.3 g. It can be seen that the normalized power density of the PEHS (0.216 μW·g^−1^·Hz^−1^·cm^−3^) was 34 times higher than the normalized power density of the SPC at the same acceleration level of 0.3 g.

## 5. Conclusions

In summary, this work presented the design, fabrication, and experimental testing of a MEMS PEHS. As a parallel structure, the PEHS consisted of a piezoelectric cantilever, a stainless-steel S-shaped cantilever with proof mass, and supporting frames. By employing the parallel-cantilever structure, the bottom low-frequency SSC would impact on the top SPC during vibration and realize a frequency up-conversion. The piezoelectric cantilever chip used in the harvester was fabricated using a PZT thick film MEMS fabrication process. Furthermore, the key techniques during fabrication were Cu-Sn-Cu eutectic bonding, mechanical lapping, and electrode layer etching. Experimental results showed that the SPC vibrated at an acceleration of 0.3 g could generate the maximum output power of 0.15 μW at the resonant frequency of 1012 Hz, and the normalized power density was 0.006 μW·g^−1^·Hz^−1^·cm^−3^. The output performances of the PEHS were also investigated under different initial gap distances and accelerations. Under a gap distance of 0.9 mm, the normalized power density of the PEHS was measured to be 0.216 μW·g^−1^·Hz^−1^·cm^−3^ at an acceleration of 0.3 g and resonant frequency of 40 Hz, which was much higher than that of the SPC. Moreover, the half-bandwidth of the PEHS broadened to 5 Hz due to the collision between SSC and SPC. It was proven that combining the PEHS with a frequency up-conversion mechanism can increase the output power under low frequency and low acceleration vibrations.

## Figures and Tables

**Table 1:** Structural parameters and material properties of the straight piezoelectric cantilever.

Parameters	Description	Value
*L*	Total length of the chip	22 mm
*W*	Total width of the chip	21 mm
*L_b_*	Length of the cantilever beam	15 mm
*L_m_*	Length of the Si proof mass	5 mm
*W_m_*	Width of the Si proof mass	14 mm
*L_n_*	Length of the nickel proof mass	15 mm
*W_n_*	Width of the nickel proof mass	5 mm
*w_s_*	Width of the S-shaped cantilever	1.5 mm
*t_te_*	Thickness of the top Cu electrode	1 μm
*t_p_*	Thickness of the PZT layer	65 μm
*t_be_*	Thickness of the bottom electrode	9 μm
*t_s_*	Thickness of the Si substrate	200 μm
*t_m_*	Thickness of the Si proof mass	300 μm
*t_n_*	Thickness of the nickel proof mass	3 mm
*t_l_*	Thickness of the stainless-steel cantilever	100 μm

## References

[1] Jung I., Shin Y.-H., Kim S., Choi J., Kang C.-Y. (2017). Flexible piezoelectric polymer-based energy harvesting system for roadway applications. Appl. Energy.

[2] Hou C., Chen T., Li Y., Huang M., Shi Q., Liu H., Sun L., Lee C. (2019). A rotational pendulum based electromagnetic/triboelectric hybrid-generator for ultra-low-frequency vibrations aiming at human motion and blue energy applications. Nano Energy.

[3] Wu J., Yuan S., Zhao X., Yin Y., Ye W. (2007). A wireless sensor network node designed for exploring a structural health monitoring application. Smart Mater. Struct..

[4] Ahmad I., Khan F.U. (2018). Multi-mode vibration based electromagnetic type micro power generator for structural health monitoring of bridges. Sens. Actuators, A..

[5] Shaikh F.K., Zeadally S. (2016). Energy harvesting in wireless sensor networks: A comprehensive review. Renewable Sustainable Energy Rev..

[6] Zhao X., Cai J., Guo Y., Li C., Wang J., Zheng H. (2018). Modeling and experimental investigation of an AA-sized electromagnetic generator for harvesting energy from human motion. Smart Mater. Struct..

[7] Liu H., Hou C., Lin J., Li Y., Shi Q., Chen T., Sun L., Lee C. (2018). A non-resonant rotational electromagnetic energy harvester for low-frequency and irregular human motion. Appl. Phys. Lett..

[8] Liu H., Lee C., Kobayashi T., Tay C.J., Quan C. (2012). Piezoelectric MEMS-based wideband energy harvesting systems using a frequency-up-conversion cantilever stopper. Sens. Actuators, A.

[9] Fan K., Tan Q., Zhang Y., Liu S., Cai M., Zhu Y. (2018). A monostable piezoelectric energy harvester for broadband low-level excitations. Appl. Phys. Lett..

[10] Wang J., Wen S., Zhao X., Zhang M., Ran J. (2016). Piezoelectric Wind Energy Harvesting from Self-Excited Vibration of Square Cylinder. J. Sensors.

[11] Zi Y., Lin L., Wang J., Wang S., Chen J., Fan X., Yang P.K., Yi F., Wang Z.L. (2015). Triboelectric-pyroelectric-piezoelectric hybrid cell for high-efficiency energy-harvesting and self-powered sensing. Adv. Mater..

[12] Yoo D., Park S.-C., Lee S., Sim J.-Y., Song I., Choi D., Lim H., Kim D.S. (2019). Biomimetic anti-reflective triboelectric nanogenerator for concurrent harvesting of solar and raindrop energies. Nano Energy.

[13] Zhang Y., Wang T., Luo A., Hu Y., Li X., Wang F. (2018). Micro electrostatic energy harvester with both broad bandwidth and high normalized power density. Appl. Energy.

[14] Naito Y., Uenishi K. (2019). Electrostatic MEMS Vibration Energy Harvesters inside of Tire Treads. Sensors.

[15] Han D., Yun K.-S. (2014). Piezoelectric energy harvester using mechanical frequency up conversion for operation at low-level accelerations and low-frequency vibration. Microsyst. Technol..

[16] Tang G., Yang B., Liu J., Xu B., Zhu H., Yang C. (2014). Development of high performance piezoelectric d33 mode MEMS vibration energy harvester based on PMN-PT single crystal thick film. Sens. Actuators, A.

[17] Zawada T., Hansen K., Lou-Moeller R., Ringgaard E., Pedersen T., Thomsen E.V. (2010). High-performance piezoelectric thick film based energy harvesting micro-generators for MEMS. Procedia Eng..

[18] He X., Wen Q., Lu Z., Shang Z., Wen Z. (2018). A micro-electromechanical systems based vibration energy harvester with aluminum nitride piezoelectric thin film deposited by pulsed direct-current magnetron sputtering. Appl. Energy.

[19] Elfrink R., Kamel T.M., Goedbloed M., Matova S., Hohlfeld D., van Andel Y., van Schaijk R. (2009). Vibration energy harvesting with aluminum nitride-based piezoelectric devices. J. Micromech. Microeng..

[20] Joshi S., Nayak M.M., Rajanna K. (2014). Effect of post-deposition annealing on transverse piezoelectric coefficient and vibration sensing performance of ZnO thin films. Appl. Surf. Sci..

[21] Md Ralib A.A., Nordin A.N., Salleh H., Othman R. (2012). Fabrication of aluminium doped zinc oxide piezoelectric thin film on a silicon substrate for piezoelectric MEMS energy harvesters. Microsyst. Technol..

[22] Tanaka K., Konishi T., Ide M., Sugiyama S. (2006). Wafer bonding of lead zirconate titanate to Si using an intermediate gold layer for microdevice application. J. Micromech. Microeng..

[23] Yang B., Zhu Y.B., Wang X.Z., Liu J.Q., Chen X., Yang C.S. (2014). High performance PZT thick films based on bonding technique for *d_31_* mode harvester with integrated proof mass. Sens. Actuators, A.

[24] Tang G., Liu J., Yang B., Luo J., Liu H., Li Y., Yang C., He D., Dao V.D., Tanaka K. (2012). Fabrication and analysis of high-performance piezoelectric MEMS generators. J. Micromech. Microeng..

[25] Lei A., Xu R., Thyssen A., Stoot A.C., Christiansen T.L., Hansen K., Lou-Moller R., Thomsen E.V., Birkelund K. MEMS-based thick film PZT vibrational energy harvester. Proceedings of the 2011 IEEE 24th International Conference on Micro Electro Mechanical Systems.

[26] Cui Y., Zhang Q., Yao M., Dong W., Gao S. (2015). Vibration piezoelectric energy harvester with multi-beam. AIP Adv..

[27] Licheng D., Zhiyu W., Xingqiang Z., Chengwei Y., Guoxi L., Jike M. (2014). High Voltage Output MEMS Vibration Energy Harvester in d_31_ Mode With PZT Thin Film. J. Microelectromech. Syst..

[28] Xu R., Lei A., Dahl-Petersen C., Hansen K., Guizzetti M., Birkelund K., Thomsen E.V., Hansen O. (2012). Screen printed PZT/PZT thick film bimorph MEMS cantilever device for vibration energy harvesting. Sens. Actuators, A.

[29] Wang Z., Miao J., Tan C.W. (2009). Acoustic transducers with a perforated damping backplate based on PZT/silicon wafer bonding technique. Sens. Actuators, A.

[30] Janphuang P., Lockhart R., Uffer N., Briand D., de Rooij N.F. (2014). Vibrational piezoelectric energy harvesters based on thinned bulk PZT sheets fabricated at the wafer level. Sens. Actuators, A.

[31] Lin S.-C., Wu W.-J. (2013). Fabrication of PZT MEMS energy harvester based on silicon and stainless-steel substrates utilizing an aerosol deposition method. J. Micromech. Microeng..

[32] Li G., Yi Z., Hu Y., Liu J., Yang B. (2018). High-performance low-frequency MEMS energy harvester via partially covering PZT thick film. J. Micromech. Microeng..

[33] Ju S., Ji C.-H. (2018). Impact-based piezoelectric vibration energy harvester. Appl. Energy.

[34] Pillatsch P., Yeatman E.M., Holmes A.S. (2014). A piezoelectric frequency up-converting energy harvester with rotating proof mass for human body applications. Sens. Actuators, A.

[35] Galchev T.V. (2008). Energy Scavenging From Low Frequency Vibrations. IEEE Sens. J..

[36] Galchev T., Aktakka E.E., Najafi K. (2012). A Piezoelectric Parametric Frequency Increased Generator for Harvesting Low-Frequency Vibrations. J. Microelectromech. Syst..

[37] Umeda M., Nakamura K., Ueha S. (1997). Energy Storage Characteristics of a Piezo-Generator using Impact Induced Vibration. Jpn. J. Appl. Phys..

[38] Halim M.A., Park J.Y. (2014). Theoretical modeling and analysis of mechanical impact driven and frequency up-converted piezoelectric energy harvester for low-frequency and wide-bandwidth operation. Sens. Actuators, A.

[39] Jung S.-M., Yun K.-S. (2010). Energy-harvesting device with mechanical frequency-up conversion mechanism for increased power efficiency and wideband operation. Appl. Phys. Lett..

[40] Andò B., Baglio S., Marletta V., Bulsara A.R. (2019). Modeling a Nonlinear Harvester for Low Energy Vibrations. IEEE Trans. Instrum. Meas..

[41] Yao L.Q., Zhang J.G., Lu L., Lai M.O. (2004). Nonlinear Dynamic Characteristics of Piezoelectric Bending Actuators Under Strong Applied Electric Field. J. Microelectromech. Syst..

[42] Gu L., Livermore C. (2011). Impact-driven, frequency up-converting coupled vibration energy harvesting device for low frequency operation. Smart Mater. Struct..

[43] Chen S., Ma L., Chen T., Liu H.C., Sun L.N., Wang J.X. (2017). Modeling and verification of a piezoelectric frequency-up-conversion energy harvesting system. Microsyst. Technol..

